# Decreased susceptibility to vancomycin and other mechanisms of resistance to antibiotics in *Staphylococcus epidermidis* as a therapeutic problem in hospital treatment

**DOI:** 10.1038/s41598-023-40866-3

**Published:** 2023-08-21

**Authors:** Magdalena Szemraj, Paulina Glajzner, Monika Sienkiewicz

**Affiliations:** Department of Pharmaceutical Microbiology and Microbiological Diagnostic, Medical University of Lodz, Lodz, Poland

**Keywords:** Antimicrobials, Bacteria, Clinical microbiology

## Abstract

Multidrug-resistant coagulase-negative staphylococci represent a real therapeutic challenge. The aim of the study was to emphasize the importance of heteroresistance to vancomycin presence in methicillin-resistant strains of *S. epidermidis*. The research comprised 65 strains of *S. epidermidis*. Heteroresistance to vancomycin was detected with the use of the agar screening method with Brain Heart Infusion and a population profile analysis (PAP test). In addition, types of cassettes and genes responsible for resistance to antibiotics for 22 multidrug resistant strains were determined. Our investigations showed that 56 of 65 *S. epidermidis* strains were phenotypically resistant to methicillin. The tested strains were mostly resistant to erythromycin, gentamicin, clindamycin, and ciprofloxacin. Six strains showed decreased susceptibility to vancomycin and their heterogeneous resistance profiles were confirmed with the PAP test. All tested multi-resistant strains exhibited the *mecA* gene. More than half of them possessed type IV cassettes. *ant(4′)-Ia* and *aac(6′)/aph(2′′), ermC* and *tetK* genes were most commonly found. The described phenomenon of heteroresistance to vancomycin in multidrug resistant bacteria of the *Staphylococcus* genus effectively inhibits a therapeutic effect of treatment with this antibiotic. That is why it is so important to search for markers that will enable to identify heteroresistance to vancomycin strains under laboratory conditions.

## Introduction

The role of coagulase-negative staphylococci (CoNS) as etiological agents of serious infection is increasing. They are one of the main causes of bacteremia in patients with indwelling medical devices, mainly induced by use of catheter and connected with vascular accesses^1–4^. It is often difficult to determine the clinical relevance of isolates obtained from patient samples because it requires differentiating between isolates being causative agents of infection and those that result from sample contamination. Recently, CoNS, mainly *S. epidermidis*, have been a real therapeutic challenge as the strains produce biofilm, which makes them even more resistant to many drugs^5,6^.

Occurrence of biofilm that adheres to surfaces and evades the host’s immune system is considered the primary cause of *S. epidermidis* virulence^7,8^. The ability to produce extracellular polysaccharide, called polysaccharide intercellular adhesin (PIA), is also regarded as one of most important factors^9^. It was reported that *S. epidermidis* also produces other proteins that may be crucial in biofilm formation, such as accumulation-associated protein (Aap) or Bhp protein, the latter being a homolog of biofilm-associated protein (Bap), found in *Staphylococcus aureus*^10,11^. Bacteria concentrated in biofilms are more resistant to antibiotics than their planktonic forms resulting in therapeutic failures.

Methicillin-resistant CoNS, characterized by clinical resistance to β-lactam antibiotics, are particularly dangerous^12^. This resistance is conditioned by the presence of the *mecA* gene encoding penicillin-binding protein 2a (PBP2a), which is located on mobile genetic elements, called the staphylococcal chromosome cassette *mec* (SCC*mec*)^13^. Among *S. epidermidis* strains, the SCC*mec* type IV is most frequently detected^14,15^. Isolates resistant to β-lactam antibiotics are often also resistant to other classes of antibiotics, most commonly to aminoglycosides, macrolides, and lincosamides^16^. This fact makes treatment of CoNS-induced infections increasingly difficult. Moreover, these bacteria, accumulating numerous antibiotic resistance genes, make up a reservoir of genes available for other bacterial species, including *S. aureus*^13^.

Glycopeptides, mainly vancomycin, are administered in treatment of methicillin-resistant CoNS-induced infections^17^. In recent years, resistance of gram-positive bacteria to vancomycin associated with transfer of *van* genes from enterococci has been reported^18^. Failure of vancomycin therapy may be also caused by heteroresistance of bacterial cells. The reason for this phenomenon are structural changes in cells, mainly their increased cell wall thickness. In addition, the bacteria produce peptidoglycan containing a large number of free D-Ala-D-Ala residues. Due to these changes fewer vancomycin molecules reach their target sites, which reduces the activity of the antibiotic^19^.

Considering the increasing resistance of coagulase-negative staphylococci to the majority of available antibiotics, and thus increasing difficulties in treatment of human infections induced by multi-resistant strains. It can be concluded that further studies are necessary to identify the mechanisms of transmission of antibiotic resistance genes in this group of bacteria. The aim of the study was to emphasize the importance of heteroresistance to vancomycin in methicillin-resistant *S. epidermidis* strains isolated hospitalized infected patients. An analysis of antibiotic susceptibility and selected resistance genotypes was also conducted.

## Materials and methods

### Tested strains

The research was conducted on 65 *Staphylococcus epidermidis* strains derived from patients of hospitals in Poland in 2018. The strains were isolated from blood, wounds and many different organs e.g. the eye, peritoneum, and urethra. The identification was performed with the use of PCR by amplification of species-specific DNA fragments^20^. *Staphylococcus epidermidis* ATCC 12228 was used as a control.

### Detection of antibiotic resistance phenotype

Resistance to antibiotics was determined by the disc-diffusion method (using Becton Dickinson discs). The following antibiotics were applied: cefoxitin (FOX-30), clindamycin (CC-2), erythromycin (E-15), tetracycline (TE-30), gentamicin (GM-10), ciprofloxacin (CIP-5), tigecycline (TGC-15), linezolid (LZD-30), cotrimoxazole (SXT-1.25/23.75), rifampicin (RA-5), and fusidic acid (FA-10). Results were interpreted in accordance to EUCAST guidelines (The European Committee on Antimicrobial Susceptibility Testing)^21^. The authors applied the CLSI guidance (Clinical & Laboratory Standards Institute) for LZD-30. MLS_B_ resistance mechanisms were detected by the D-test. Resistance to erythromycin and clindamycin was interpreted as a constitutive type of the MLS_B_ mechanism (cMLS_B_), while resistance to erythromycin and existence of a flattening zone around the disc with clindamycin from the erythromycin side was interpreted as presence of inducible resistance (iMLS_B_). For methicillin-resistant *Staphylococcus epidermidis* (MRSE), the susceptibility to teicoplanin, vancomycin and daptomycin was determined with the broth microdillution method according to EUCAST guidelines. Dilutions of the antibiotics were applied at the following concentrations: 16–0.25 mg/L. The strains exhibited reduced susceptibility to vancomycin for the MIC value 1–2 mg/L. *Staphylococcus aureus* ATCC 29213 was used as a control strain.

### Detection of vancomycin heteroresistance

In order to detect vancomycin heteroresistance in *S. epidermidis* strains, Brain Heart Infusion (BHI) screen agar was used^22,23^. The suspensions of 0.5 McFarland density were prepared with the use of night bacterial cultures cultivated on blood agar. Four drops of 10 μL from each suspension were put on a BHI agar plate (Oxoid) with 4 mg/L vancomycin (Sigma). The plates were incubated at 37 °C for 48 h. Mature colonies incubated in each drop were counted after the incubation. The strain was marked as heterogeneous vancomycin *S. epidermidis* (hVISE) if at least one drop contained minimum two colonies.

### Population analysis profile (PAP) for vancomycin heteroresistance

Vancomycin heteroresistance in subpopulations was detected for the strains that grow on a BHI agar plate with 4 μg/mL vancomycin in accordance with the method described by Kim et al.^24^ For this purpose, serial tenfold dilutions of sterile saline bacterial suspensions of 0.5 McFarland density were prepared. Then, 100 μL was placed on BHI agar plates with vancomycin at concentrations of 2–16 mg/L. The plates were incubated at 35 °C for 48 h. Afterwards, the authors counted the colonies that grew in particular concentration of vancomycin. Heterogeneous vancomycin susceptible *Staphylococcus aureus* (hVSSA) strain ATCC 29213 was the negative control, while the heterogeneous vancomycin intermediate resistant *Staphylococcus aureus* (hVISA) strain Mu3 (ATCC 700698) was the positive control. The procedure was repeated three times.

### DNA isolation for methicillin-resistant strains

Multi-resistant strains (resistant to at least four antibiotics) were selected for genetic studies. DNA was isolated with the use of the Genomic Micro AX Staphylococcus Gravity set (A&A Biotechnology) in accordance to the manufacturer’s protocol.

### Detection of resistance genes by the PCR method

Genes conditioning the resistance to β-lactam antibiotics (*mecA*)^25^, aminoglycosides (*aac(6′)/aph(2″)*, *aph(3′)-IIIa*, *ant(4′)-Ia*)^26,27^, macrolides and lincosamides (*ermA*, *ermB*, *ermC, msrA, msrB, mphC, lnuA*)^28,29^, as well as tetracyclines (*tetK, tetL, tetM, tetO*)^30–32^ were identified by PCR. The strains demonstrated decreased susceptibility to vancomycin (MIC = 1–2 mg/L) also v*anA* and *vanB* genes were detected^33^. Obtained PCR reaction products were split electrophoretically in 1% agarose gel with 1 µl Midori Green DNA Advance Stain (NIPPON Genetics Europe GmbH, Germany) supplement.

### SCCmec cassette typing by PCR

For strains harboring the *mecA* gene, SCC*mec* cassette typing was performed according to the method described by Kondo et al. and Zhang et al. *AB1, AB2, AB3* and *C ccr* gene complex types and *A, B* and *C mec* gene complex classes were identified^34,35^. The *ccrAB4* gene was marked with the method described by Oliveira et al. with Zhang et al. with modification^35,36^. Cassettes with combination of *ccr* and/or *mec* genes undescribed before, in accordance to the rules set by International Working Group on the Classification of Staphylococcal Cassette Chromosome Elements, were considered potentially new types of cassettes^37^.

### Statistical analysis

A relationship between results obtained for the cassette type and phenotypic antibiotic resistance and gene presence was determined with the use of the chi-squared test. *p* < 0.05 was adopted as significant. STATISTICA 13.1PL software was used (StatSoft 2016, Poland) for the purpose of statistical analysis.

### Ethics approval and consent to participate

Not applicable. In the study, informed consent was not required as the isolates included in the study were obtained as a result of standard medical care. Patients’ identity as well as all their personal information were confidential.

## Results

Our studies showed that 56 of 65 *S. epidermidis* strains were phenotypically resistant to methicillin, what suggests their resistance to all β-lactams. They were mostly resistant to erythromycin, gentamicin, clindamycin and ciprofloxacin. Single strains were resistant to fusidic acid, rifampicin and tigecycline. All of them were also methicillin-resistant. Resistance to linezolid was not observed. 22 of methicillin-resistant strains were multi-resistant and exhibited resistance to at least four of the antibiotics used in the studies. The resistance to antibiotics of all tested strains is presented in Table 1.

**Table 1:** Antibiotic resistance in tested *S. epidermidis*.

Antibiotics	% of resistant strains
FOX-30	86
E-15	60
CC-2	43
GM-10	48
TE-30	35
CIP-5	38
TGC-15	3
LZD-30	0
SXT-23.75/1.25	34
RA-5	8
FA-10	9

FOX-30—cefoxitin, E-15—erythromycin, CC-2—clindamycin, GM-10—gentamicin, TE-30—tetracycline, CIP-5—ciprofloxacin, TGC-15—tigecycline, LZD-30—linezolid, SXT-23.75/1.25—cotrimoxazole, RA-5—rifampicin, FA-10—fusidic acid.

**Figure 1:**
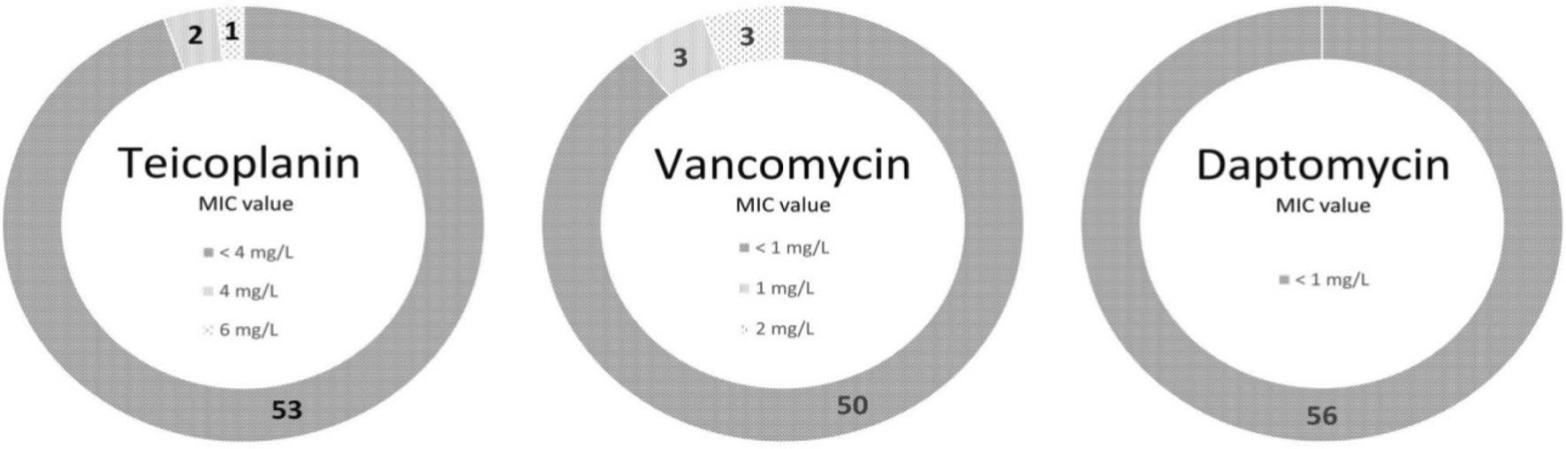
Teicoplanin, vancomycin and daptomycin MICs in tested methicillin-resistant *S. epidermidis*.

With regards to MRSE, MIC values for teicoplanin, vancomycin and daptomycin were determined with the microdillution broth method. Results are presented in Fig. 1.

**Figure 2:**
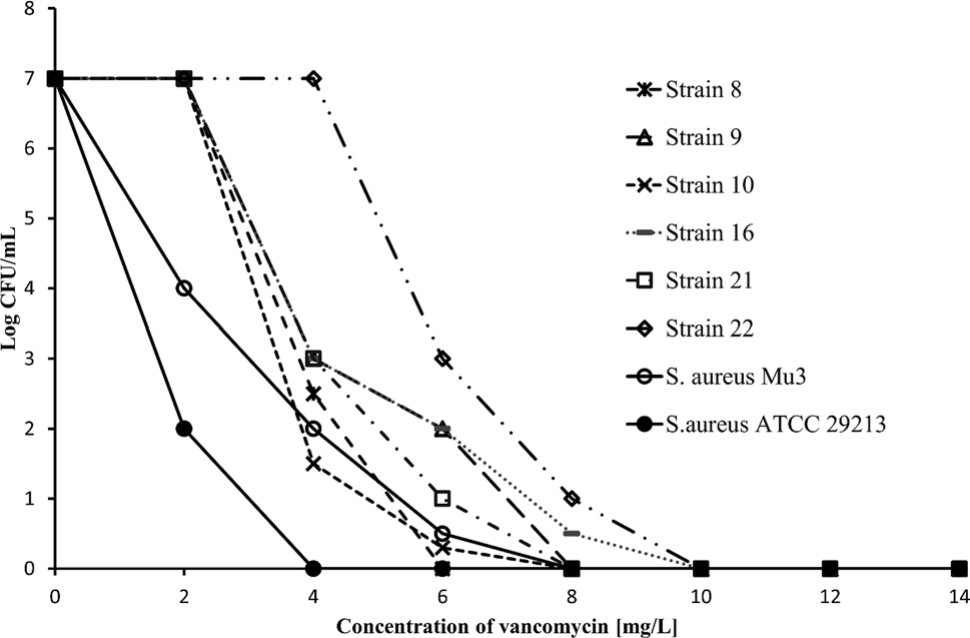
Analysis of population analysis profiles (PAPs) for six strains of *S. epidermidis* with reduced susceptibility to vancomycin.

Only one *S. epidermidis* strain was resistant to teicoplanin (MIC = 6 mg/L). According to EUCAST guidelines, all strains were sensitive to vancomycin and daptomycin. Vancomycin MIC for the majority of strains was below 1 mg/L. However the growth of three strains was inhibited with 2 mg/L vancomycin and three with 1 mg/L of this antibiotic. These strains were characterized by decreased susceptibility to vancomycin, and grew on a BHI agar plate with 4 mg/L of vancomycin. Their heterogeneous resistance profiles were confirmed in the PAP test. Subpopulations of four strains grew on BHI agar with 6 mg/L, while two grew with the application of 8 mg/L vancomycin. PAP test results for the tested strains of *S. epidermidis* and control strains (*S. aureus* ATCC 29,213, susceptible to vancomycin and Mu3, hVISA) are shown in Fig. 2.

Strains with reduced susceptibility to vancomycin were characterized by absence of *van* genes. Cassette types were determined and genes responsible for resistance to β-lactams, aminoglycosides, tetracyclines and the MLS_B_ mechanism were identified in 22 multidrug-resistant strains. All tested strains, phenotypically resistant to methicillin, possessed the *mecA* gene. Type IV cassettes were detected in more than half of them, whereas type V cassettes were found in four strains. Presence of cassette types are shown in Table 2.

**Table 2:** Types of cassettes detected in tested *S. epidermidis* strains.

SCC*mec* cassette								Cassette type	No. of isolates
*ccr* complex					*mec* complex				
AB1	AB2	AB3	AB4	C	A	B	C		
−	+	−	−	−	−	+	−	IV	12
−	−	−	−	+	−	−	+	V	4
−	−	+	−	−	+	−	−	III	2
−	+	−	−	+	+	−	−	II	1
−	−	−	−	−	+	−	−	N	1
−	−	−	−	−	−	−	+	N	1
−	−	−	−	+	−	−	−	N	1

**Figure 3:**
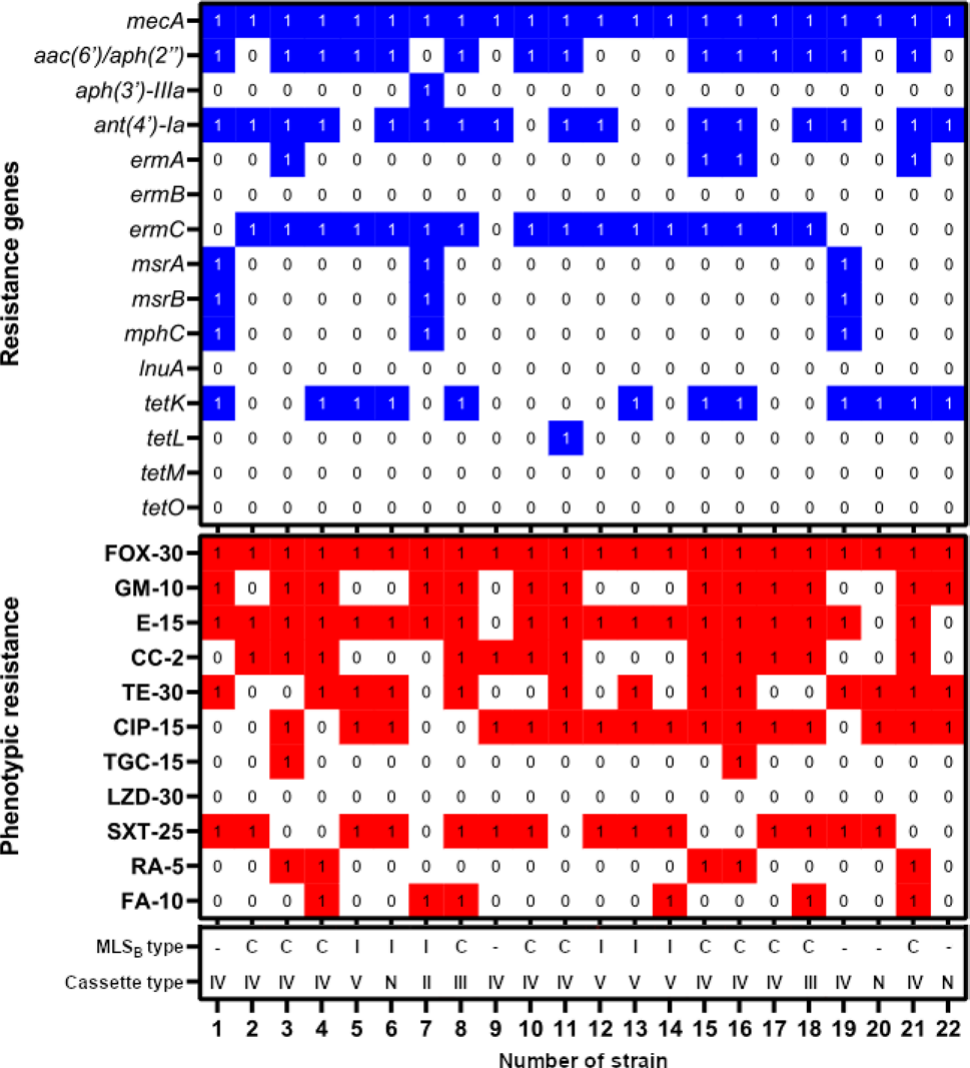
Antibiotic resistance phenotypic profile and resistance genes in 22 multi-resistant strains of *S. epidermidis*. FOX-30—cefoxitin, E-15—erythromycin, CC-2—clindamycin, GM-10—gentamicin, TE-30—tetracycline, CIP-5—ciprofloxacin, TGC-15—tigecycline, LZD-30—linezolid, SXT-1.25/23.75—cotrimoxazole, RA-5—rifampicin, FA-10—fusidic acid; I—inductive mechanism MLS_B_ (iMLS_B_), C—constitutive mechanism MLS_B_ (cMLS_B_).

The phenotypic profile of antibiotic resistance and resistance genes, including the type of SCC*mec* cassette in 22 multi-resistant strains, is shown in Fig. 3.

The following genes encoding resistance to aminoglycosides were the most common: *ant(4')-Ia* and *aac(6')/aph(2'')*, observed respectively in 73% and 64% of the tested strains. The presence of the *aac(6')/aph(2")* gene was statistically significant for strains with type IV cassette (*p* = 0.03). Five strains with at least one of the genes, did not demonstrate phenotypic resistance to gentamicin.

The *ermC* gene was present in 16 strains, all of which were characterized by a constitutive or inductive MLS_B_ mechanism. One cMLS_B_ strain possessed only the *ermA* gene. This gene was present in three strains, but together with *ermC*. The *mphC, msrA and msrB* genes were present in three strains, and were always observed together. Two strains having these genes were erythromycin resistant, and in one strain, they occurred together with the *ermC* gene.

All strains with *tet* genes were resistant to tetracycline. The *tetK* gene was detected in 12 out of 13 resistant strains. Five strains were rifampicin resistant. All had type IV cassette, which was statistically significant (*p* = 0.02). There was also a statistically significant relationship between the presence of this cassette and clindamycin resistance (*p* = 0.02) and the presence of this cassette and the *ermA* gene (*p* = 0.04). Two strains were resistant to nine tested antibiotics, which is an alarming number from the therapeutic point of view. Both the strains had type IV cassette and at least five detectable genes. Besides, they demonstrated reduced sensitivity to vancomycin. Table 3 shows antibiotic resistance profiles and occurrence of detected genes in six strains characterized by vancomycin heteroresistance. The table also includes the origin of the isolate and the clinical department where the infected patient was hospitalized.

**Table 3:** Characteristics of vancomycin hetero-resistant strains.

Number of the strain		Isolation site	Clinical department	Growth on BHI medium with vancomycin (mg/L)	Phenotypic resistance	Type of cassettes	Resistance genes
Vancomycin MIC = 1 mg/L							
8	Blood		Neurology	6	FOX, CC, E, GM, TE, SXT, FA	III	*mecA, aac(6′)/aph(2″), ant(4′)-Ia, ermC, tetK*
9	Blood		Intensive care unit	6	FOX, CC, CIP, SXT	IV	*mecA, ant(4′)-Ia*
10	BBlood		Intensive care unit	6	FOX, CC, E, GM, CIP, SXT	IV	*mecA, aac(6′)/aph(2″), ermC,*
Vancomycin MIC = 2 mg/L							
16	Blood		Hematology	8	FOX, E, CC, GM, TE, CIP, SXT, TGC, RA	IV	*mecA, aac(6′)/aph(2″), ant(4′)-Ia, ermA, ermC, tetK*
21	Blood		Hematology	6	FOX, E, CC, GM, TE, CIP, SXT, RA, FA	IV	*mecA, aac(6′)/aph(2″), ant(4′)-Ia, ermA, tetK*
22	Blood		Hematology	8	FOX, GM, TE, CIP	N	*mecA, ant(4′)-Ia, tetK*

FOX-30—cefoxitin, E-15—erythromycin, CC-2—clindamycin, GM-10—gentamicin, TE-30—tetracycline, CIP-5—ciprofloxacin, TGC-15—tigecycline, SXT-1.25/23.75—cotrimoxazole, RA-5—rifampicin, FA-10—fusidic acid;

## Discussion

Presently, more and more attention is being paid to the problem of increasing resistance in bacteria. Not only does it concern pathogenic bacteria, but also opportunistic pathogens that cause infections mainly in immunocompromised people. Such bacteria include *S. epidermidis*, which causes serious problems to hospitalized people who undergo invasive procedures, as well as newborns.

In our work, we studied 65 strains of *S. epidermidis* which induced infection in hospitalized people. 86% of these tested strains were methicillin-resistant, of which 39% were resistant to at least four antibiotics. This problem is also pointed out in works of other researchers in which the percentage of methicillin-resistant strains was comparable to that we observed or even higher^38–40^. The strains tested in our investigations were mostly resistant to erythromycin, cefoxitin, gentamicin, and clindamycin and the values were the following: 60%, 56%, 48%, 43% respectively. Macrolide resistance was the most common. A similar result was reported by Skiba-Kurek et al.^38^. Of the 100 of *S. epidermidis* strains they tested, 67% were resistant to erythromycin. The percentage of resistant strains observed in studies of Mirzaei et al. was even higher—as much as 80% of the tested strains appeared to be resistant to erythromycin^39^. Increased resistance to macrolides is a consequence of their widespread application in treatment of infections caused by gram-positive bacteria^41^. In our studies, almost half of the strains were resistant to gentamicin. In the studies presented by Wojtyczka et al., the resistance of tested *S. epidermidis* strains that were isolated from nosocomial infections, was below 4%. Nevertheless, in other studies this resistance was much higher^42–44^. Therefore, it is particularly important to emphasize that, according to EUCAST recommendations, gentamicin should be administered only in combination therapy^21^. Sensitivity to glycopeptides and daptomycin was determined for MRSE strains. Vancomycin is the first line antibiotic used to treat severe infections caused by methicillin-resistant staphylococci^17^. Daptomycin can be used in therapies in which vancomycin appears to be ineffective^45^. All the strains that we tested were susceptible to vancomycin and daptomycin according to EUCAST recommendations. However, six strains demonstrated reduced susceptibility to vancomycin. Moreover, these strains exhibited heterogeneous growth in the PAP test and could grow on a medium containing 6–8 mg/L vancomycin. Mashaly et al. or Nunes et al., who studied this mechanism in CoNS, showed that prior treatment with vancomycin may result in occurrence of a hetero-resistant phenotype^19,23^.

Its mechanism has not been fully recognised so far. It does not require the presence of the *van* genes responsible for the resistance to vancomycin^46^. It was confirmed by our studies as we didn’t detect such genes in the decreased susceptibility to vancomycin strains tested by us. For such strains, vancomycin therapy may turn out to be ineffective, although, according to recommendations, they are supposed to be susceptible to this antibiotic. As a result of increasing number of the infections caused by CoNS, decreased susceptibility of these bacteria to vancomycin may account for serious problem in hospital treatment that decreases therapeutic possibilities. This phenomenon was highlighted in many studies concerning severe infections caused by different species of CoNS^23,47,48^.

All tested strains, that were phenotypically resistant to methicillin, possessed the *mecA* gene. Type IV cassettes were detected in more than half of the strains. In the case of CoNS, the diversity of cassettes is very large and may result from sources of strain isolation^49,50^. The presence of type I and III cassettes is attributed to isolates of hospital origin, while the presence of type IV and V cassettes to isolates coming from CA-MRSE (community-associated methicillin-resistant *S. epidermidis*). Additionally, existence of these particular types of cassettes may be influenced by the species from which they are isolated. Type IV cassettes are often found in *S. epidermidis*. Moreover, this cassette has been isolated for the first time from these species^51^. The analysis of the cassettes existence in *S. epidermidis* over the recent years indicates that it was the type IV cassettes that were the most common isolated in such species^14,15,48,52^. Also types I, II, III or V cassettes were detected in *S. epidermidis*^15,53,54^. Studies of Havaei et al., based on *S. epidermidis* from hospitalized patients revealed the presence of type III cassettes in 36% of the tested strains^15^. Also Martins et al. most often identified type III cassettes (50%) in their studies on *S. epidermidis*. Moreover, 32.8% of the strains had type I cassettes^55^. In our studies, type III cassettes was detected in only two strains, while type I cassettes was not found in any of the tested strains. Type II and III cassettes often contain aminoglycoside, macrolide or tetracycline resistance genes in their structure, which was confirmed in studies of Szczuka et al. Strains of *S. epidermidis* that they tested possessed type III cassettes and were characterised by much greater resistance to non-β-lactam antibiotics than those with type IV cassettes^56^. In our studies, a correlation between type IV cassettes and resistance to clindamycin and rifampicin was observed. Moreover, there was also a correlation between the presence of such cassettes and existence of *aac*(6*'*)/*aph*(2*''*) and *ermA* genes. In the tested methicillin-resistant *S. epidermidis*, described by Teodoro et al., the *ermC* gene was located mainly on SCC*mec* IV or non-typeable cassettes^57^.

Macrolides, lincosamides and streptogramins B have a similar mechanism of action leading to cross-resistance (MLS_B_), which is more and more frequently described in different groups of bacteria. The *erm* family genes is responsible for this mechanism. They encode one methylase which is responsible for the methylation of the adenine 23S rRNA ribosomal subunit. The *ermC* gene, being the dominant gene responsible for this mechanism in CoNS, was detected in 16 strains in our investigations. This gene was present in both constitutive and inductive MLS_B_ mechanism strains. cMLS_B_ determines resistance to all MLS_B_ group of antibiotics, while iMLS_B_ stands for clinical resistance to lincosamides and streptogramins B induced by 14- and 15-membered macrolides. The *msrA* and *msrB* genes encoding active efflux are responsible for resistance to macrolides and streptogramins B (MS_B_ phenotype) in the case of the susceptibility to lincosamides. They were present in three strains and always together with the *mphC* gene encoding enzymes inactivating a specific substance^28,58^. Juda et al.^59^ in their studies described strains characterized by resistance to macrolides, lincosamides and streptogramins B possessed a more diverse genotype.

In the strains tested by us we were trying to identify *aac(6′)/aph(2″), aph(3′)-IIIa, ant(4′)-Ia* genes which are responsible for the most common aminoglycoside-modifying enzymes among the staphylococci genus. Resistance to aminoglycosides was encoded mainly by *ant(4')-Ia* and *aac(6')/aph(2)* genes. The *aac(6')-Ie-aph(2'')-Ia* gene dominated also in clinical isolates of staphylococci in studies conducted by Schmitz et al.^26^. Because of increased resistance to tetracycline we also attempted to find genes encoding the resistance to this group of antibiotics. 12 strains that we tested were characterized with presence of the *tetK* gene. This gene was also detected in nearly 60% of *S. epidermidis* strains tested by Chabi and Momtaz^60^. The authors also detected the *tetM* gene in more than 39% of strains. However, we did not such strain was detected in our studies. Two strains isolated from the blood of hospitalized patients and analyzed by us reveal how dangerous for therapeutic reasons, can be *S. epidermidis*-induced infections. They were resistant to as many as nine antibiotics and possessed at least five tested genes. Treatment of these strains with vancomycin appeared to be ineffective as they both of them demonstrated reduced susceptibility to this antibiotic.

The described phenomenon of heteroresistance to vancomycin in multidrug resistant bacteria of the *Staphylococcus* genus effectively inhibits a therapeutic effect of treatment with this antibiotic. It is difficult to determine this mechanism in a routine laboratory microbiology. Strains characterized by vancomycin heteroresistance are considered susceptible according to the method recommended by EUCAST. That is why it is so important to search for markers that will enable to identify vancomycin strains under laboratory conditions. To achieve this goal further studies are required.

## Data Availability

The datasets used and/or analyzed during the current study are available from the corresponding author on reasonable request.
